# Anti-Inflammatory Potential of *Ganoderma lucidum* Triterpenes: A Systematic Review and Meta-Analysis of Preclinical Evidence

**DOI:** 10.3390/ph19010188

**Published:** 2026-01-21

**Authors:** Rafaela Guedes Pozzobon, Renata Rutckeviski, Luíza Siqueira de Lima, Cláudia Sirlene Oliveira, Fhernanda Ribeiro Smiderle

**Affiliations:** 1 Instituto de Pesquisa Pelé Pequeno Príncipe, Av. Silva Jardim, 1632, Água Verde, Curitiba 80250-060, PR, Brazil; rafaela.pozzobon@aluno.fpp.edu.br (R.G.P.); renatarut@hotmail.com (R.R.); luizaelima04@gmail.com (L.S.d.L.); claudia.sirlene@professor.fpp.edu.br (C.S.O.); 2 Faculdades Pequeno Príncipe, Av. Iguaçu, 333, Rebouças, Curitiba 80230-020, PR, Brazil

**Keywords:** medicinal mushroom, triterpenoids, bioactivity, in vitro, in vivo

## Abstract

**Background**: *Ganoderma lucidum* triterpenes are bioactive compounds with recognized anti-inflammatory, antitumor, and immunomodulatory properties. This systematic review synthesizes evidence regarding the anti-inflammatory activity of these triterpenes based on studies from the last two decades. **Methods**: A systematic search was performed in PubMed, Medline, and Embase (2003–2025) for original in vitro and in vivo (non-clinical) studies evaluating *G. lucidum* triterpene extracts or isolated compounds. Clinical trials, reviews, and multi-species extracts were excluded. The review is registered on PROSPERO (CRD42024510982), and animal study quality was assessed using the SYRCLE Risk of Bias tool. **Findings**: From over 3000 records, 23 articles were included. Studies utilized diverse models, including macrophages, human PBMCs, and various animal strains (mice, rats, chickens). All studies reported significant anti-inflammatory effects via reduction in pro-inflammatory markers (TNF-α, IL-1β, IL-6), primarily through downregulation of MAPK and TLR-4/NF-κB signaling pathways. Meta-analysis of in vitro data confirmed significant reductions in NO levels (−3.29 [95% CI: −5.21, −1.37]; *p* = 0.0008), IL-6 (−3.51 [−4.73, −2.29]; *p* < 0.00001), and TNF-α (−2.20 [−2.93, −1.48]; *p* < 0.00001). Similar anti-inflammatory profiles were observed in vivo across hepatic and splenic tissues. **Interpretation**: Evidence consistently demonstrates the potent anti-inflammatory activity of *G. lucidum* triterpenes, highlighting their potential as therapeutic candidates for inflammatory diseases. However, the structural complexity and isomer diversity of these compounds remain significant barriers to pharmacological standardization. Future research must prioritize clinical translation by investigating compound synergism, bioavailability, and long-term toxicity profiles, which were notably absent in current non-clinical literature.

## 1. Introduction

*Ganoderma lucidum*, known as “Lingzhi” in China, “Reishi” in Japan, and “Yeongji” in Korea, is one of the most widely consumed medicinal mushrooms worldwide. It is currently cultivated on a large scale, particularly in China, and exported for the production of teas, dietary supplements, and raw materials used in edible and medicinal products [1]. *G. lucidum* has been extensively used in traditional Chinese medicine to treat a variety of conditions, including hepatitis, cancer, hypertension, hypercholesterolemia, diabetes, arthritis, and asthma, owing to its recognized beneficial effects [2,3]. However, the increasing global consumption of *G. lucidum* products has raised important concerns regarding quality control and regulatory oversight. Regulatory oversight for evaluating the quality and quantity of bioactive compounds in dietary supplements is often insufficient [4]. For example, an analysis of *G. lucidum* dietary supplements commercialized in the United States revealed that only 26.3% of the products were compliant with their label claims [4].

This macrofungus contains a wide range of bioactive compounds, including carbohydrates (mainly polysaccharides), nucleotides, steroids, fatty acids, vitamins, proteins, minerals, organic acids, glycopeptides, and triterpenes [5]. Triterpenes are secondary metabolites composed of 30 carbon atoms derived from six isoprene units and are commonly classified into tetracyclic and pentacyclic structures, which exhibit diverse pharmacological activities. The predominant triterpenes identified in *G. lucidum* are lanostane-type compounds, particularly ganoderic acids and their derivatives [6,7]. Studies investigating *G. lucidum* triterpene extracts have reported multiple biological activities, including antioxidant, anti-inflammatory, antitumor, anti-angiogenic, anti-HIV-1, immunomodulatory, hypocholesterolemic, hepatoprotective, antihistaminic, antihypertensive, antiplatelet, and complement-inhibitory effects [8,9,10].

Inflammation is a fundamental biological response of the immune system, and a tightly regulated balance between insufficient and excessive inflammation is essential for organism survival [11]. Inflammatory processes may be triggered by external agents, such as microorganisms, toxins, and physical or chemical stimuli, as well as internal factors including ischemic injury and autoimmune disorders [11]. Evidence suggests that many of the biological effects of triterpenes are mediated through immunomodulatory mechanisms [7]. In this context, macrophages have been extensively studied, as these innate immune cells play a central role in regulating inflammatory responses, tissue homeostasis, and repair [12]. Activated macrophages participate in the phagocytosis of damaged cells and invading pathogens and secrete inflammatory mediators such as tumor necrosis factor, interleukins, reactive oxygen species, nitric oxide, and prostaglandin E2 [13]. Although this activation is essential for host defense, excessive or dysregulated mediator production has been associated with the development and progression of multiple inflammatory diseases [14].

Triterpene extracts from *G. lucidum* have been shown to suppress the inflammatory responses in lipopolysaccharide (LPS)-activated murine macrophages. For instance, Dudhgaonkar et al. [15] demonstrated that a triterpene extract inhibited LPS-induced secretion of TNF-α, IL-6, nitric oxide, and PGE2 in RAW264.7 cells. Similarly, ganoderic acid C1 reduced TNF-α production in RAW264.7 cells and peripheral blood mononuclear cells from patients with asthma, an effect associated with downregulation of NF-κB and partial suppression of MAPK and AP-1 signaling pathways [16].

Despite these promising findings, important gaps remain in the literature. Active concentrations, toxicity profiles, and sub-chronic toxicity have not been comprehensively evaluated, and the chemical variability of triterpenes and other constituents in *G. lucidum* extracts has not been systematically addressed. Consequently, there is still limited consolidated evidence regarding the anti-inflammatory effects of *G. lucidum* triterpenes and their direct translational applicability.

Based on these considerations, this systematic review aims to consolidate and critically analyze the available literature on the anti-inflammatory effects of *G. lucidum* triterpenes, providing an organized framework to support future experimental and translational research.

This review specifically addresses the following question: Do *Ganoderma lucidum* triterpenes exhibit anti-inflammatory effects in cell-based and/or animal models compared with appropriate controls?

## 2. Materials and Methods

This systematic review was carried out following the Preferred Reporting Items for Systematic Reviews and Meta-Analyses (PRISMA) guidelines and its protocol was registered on the International Prospective Register of Systematic Reviews (Prospero) under the number CRD42024510982.

### 2.1. PICO Criteria

The PICO criteria (Participants/population, Intervention, Comparison and Outcome) used in the present systematic review were (a) Population: cell lines and/or animal models; (b) Intervention: triterpenes/triterpenoids or terpenes/terpenoids from *G. lucidum;* (c) Comparison: cells and/or animals treated with vehicle and/or other agent; (d) Outcome: anti-inflammatory activity.

### 2.2. Search Strategy and Study Selection

As search strategy, PubMed, Medline and Embase were used as databases, considering the following search terms: “*Ganoderma lucidum*” and “triterpenes” or “triterpenoids” or “terpenes” or “terpenoids” and “anti-inflammatory” for PubMed, and “*Ganoderma lucidum*” and “triterpenes” or “triterpenoids” and “anti-inflammatory” for Medline and Embase. The article selection stage and the evaluation of titles and abstracts were carried out by two independent reviewers (R.G.P. and R.R.). Discrepancies at all stages are resolved by a third author (F.R.S.). Review articles and duplicated articles were excluded, as well as articles in which the outcomes were not of interest. Duplicated records were removed using Rayyan (Intelligent Systematic Review—www.rayyan.ai—29 November 2025).

### 2.3. Inclusion and Exclusion Criteria

The search was focused on original non-clinical studies referring to anti-inflammatory activity of triterpene extracts and/or isolated triterpenes from *G. lucidum* in cell lines and animal models. This search was limited to a period of publication (2003–2025) and to articles published in English. Studies using animal experimental models (rats, mice, and chickens), in vitro cell line (cells induced for inflammation), as well as ex vivo blood and tissue were included. Additionally, only studies comparing the effects of triterpenes with a control group (cells/animals treated with vehicle and/or another agent) were considered. For the evaluation of anti-inflammatory activity, articles that conducted tests with inflammatory markers, such as cytokines, gene expression, and reactive oxygen species were included. Articles published in non-indexed journals and studies such as reviews, clinical trials, conference abstracts, books, book chapters and studies performed before 2003 were not eligible. Studies on total extracts of triterpenes or triterpenoids, as well as their isolates linked to other compounds or from other mushroom species, were excluded.

### 2.4. Data Extraction and Outcome Measures

Two authors (R.G.P. and R.R.) independently extracted data from the selected articles, organizing the information in MS Excel software (Microsoft Inc., Redmond, WA, USA), according to the following: intervention (type of triterpene), obtention (triterpene extraction procedure), purification procedures, chemical characterization, in vitro/in vivo models, concentration/dose of treatments, control treatment, type of inflammation induction and study design, inflammatory markers analyzed, signaling pathways, funding sources, and conflict of interest. All outcome measures, qualitative or quantitative, were recorded. Discrepancies were resolved by a third author (F.R.S.).

### 2.5. Risk of Bias Assessment

The methodological rigor of each study was evaluated by assessing the risk of bias (RoB) utilizing SYRCLE’s RoB tool designed for animal studies [17]. The assessment of the risk of bias was performed by two authors (R.G.P. and R.R.) and F.R.S. was consulted to resolve disagreements. The assessment included bias arising from the randomization process, bias due to deviations from intended intervention, bias due to missing outcome data, bias in measurement of the outcome and bias in selection of the reported result. Each analysis had three possible judgments (“low risk”, “high risk”, or “some concerns”). If the information was not described clearly, the assessment “no information” was selected. For in vitro studies, “low risk” was considered as “no applicable” for randomization process.

### 2.6. Statistical Analysis

The meta-analysis assessed the anti-inflammatory effects of the triterpenes from *Ganoderma lucidum* in LPS- or IL-1β-stimulated macrophages or other cells, focusing on the inflammatory mediator NO and the cytokines IL-6 and TNF-α. Studies that were not included in the meta-analysis and their reasons are listed in Table S1 (Supplementary Materials). Outcomes were expressed as mean ± standard deviation (SD), with 95% confidence intervals used as the measure of variance. Continuous variables were analyzed using the inverse variance method with a random-effects model, weighting means by the sample size of each study. Heterogeneity was evaluated with the I^2^ index and classified as low (<25%), mild (25–50%), moderate (50–75%), or high (>75%). Subgroup analyses were performed based on cell type. A *p*-value ≤ 0.05 was considered statistically significant. All analyses were performed using Review Manager© version 5.4.1 (Cochrane Collaboration, London, UK). When quantitative data were not provided in the text or tables, they were extracted from the figures using the free software Graph Grabber (version 2.0.2, Quintessa Software, Henley-on-Thames, UK 2017).

## 3. Results

### 3.1. Study Selection

**Figure 1:**
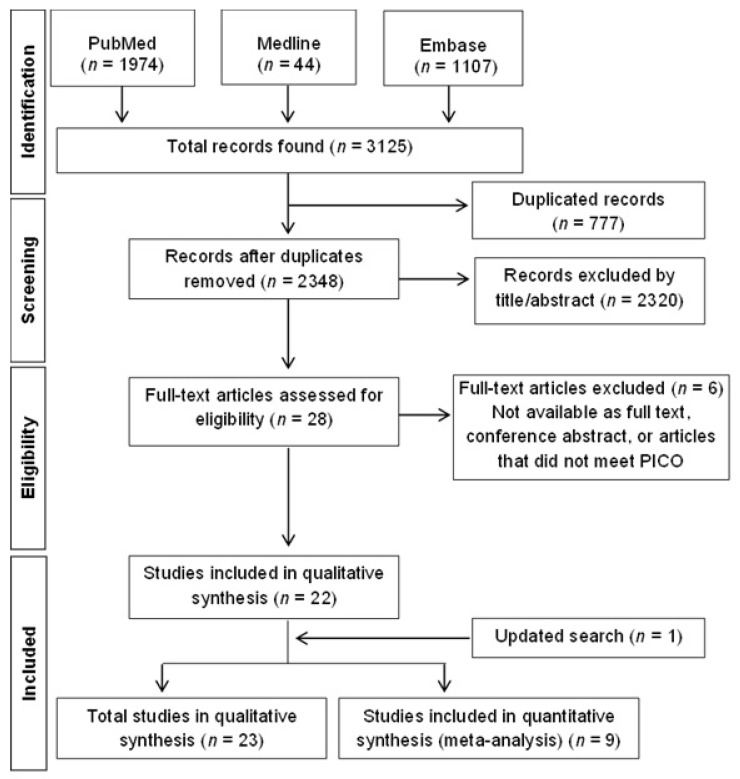
Study flow diagram.

In total, 3125 records were identified, comprising 777 duplicated records, with 2348 records screened based on abstract and title. Of those, 2320 were excluded (did not fit the criteria following title and abstract analysis). Then, a total of 28 articles were assessed for eligibility. From this, a total of 6 articles were excluded due to the following reasons: articles that did not meet the PICO criteria (*n* = 3) and other reasons (it was not possible to download the articles for full reading, *n* = 2, and conference abstract, *n* = 1). Finally, an update search was performed from 2024 to 2025, which found 1 article. Data analysis was developed in 23 studies. The flowchart of studies selected in the systematic review can be seen in Figure 1.

### 3.2. Risk of Bias Assessment Results

**Figure 2:**
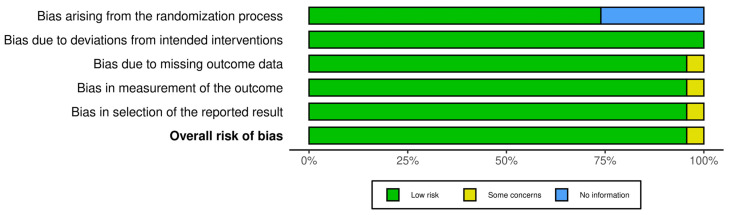
Risk of bias assessment. Evaluation of the methodological quality and assessment of the risk of bias using the SYRCLE’s RoB tool (https://mcguinlu.shinyapps.io/robvis/, 29 November 2025) designed for animal studies. The bars represent the percentage of articles found in each category.

According to the risk of bias assessment, it was possible to observe that the majority of articles presented low risk for each methodological domains assessed. However, 4% of the studies presented bias in the measurement and discussion of results (considered as some concerns). In evaluating the domain “bias arising from the randomization process,” 14 articles with in vitro studies were assessed as low risk and considered “not applicable” to the randomization process. Furthermore, among the articles showing experiments in vivo, more than 50% were evaluated as “no information” due to unclear description. According to the bias risk analysis, the selected articles will not have a negative impact on achieving the review’s objective. The risk of bias assessment is summarized in Figure 2.

### 3.3. Triterpenes from Ganoderma lucidum: Extraction and Characterization

Several triterpenes have been identified and/or isolated from the species *G. lucidum* and most of which belong to the lanostane class. Lanostane triterpenoids are a class of organic compounds characterized by a tetracyclic skeleton that serves as a precursor to many steroids. They are present in most fungal species and exhibit structural diversity along with a broad spectrum of bioactivities [18]. They can be categorized as *Ganoderma* alcohols, owing to the hydroxyl group on the lanostane moiety, or ganoderic acids, owing to the carboxyl group on the side chain [18].

According to the evaluated studies, the extraction and isolation of triterpenes from *G. lucidum* have been extensively explored, with different methodologies. The process typically begins with drying the mushrooms, followed by milling. Among the extraction methods reported, ethanol (EtOH) was the most employed solvent, followed by methanol (MeOH) and chloroform (Table 1). The choice of the solvent may produce different extract composition due to their distinct polarities and, therefore it may confer variable yields [5].

Silica gel column chromatography, partition with organic solvents of different polarities and chromatography using Sephadex LH-20 column were the preferable approaches to isolate the triterpenes. Both silica gel and Sephadex LH-20 stationary phases are vastly used to separate natural compounds such as steroids, terpenoids, lipids and small peptides. Furthermore, they are compatible and resistant to chemical changes in the presence of different organic solvents [39]. Other approaches very used on the studies of this review were HPLC, reversed phase-HPLC, semi-preparative HPLC, and TLC, which is generally used with silica gel as stationary phase, however this information was not available in some studies.

Characterization techniques were mainly based on spectroscopic methods, including nuclear magnetic resonance (NMR), mass spectrometry (MS), infrared (IR) spectroscopy, and ultraviolet (UV) spectroscopy (Table 1). The use of NMR and MS was crucial for accurate structural identification, considering that *Ganoderma* triterpenes present different radicals in the same skeleton.

**Figure 3:**
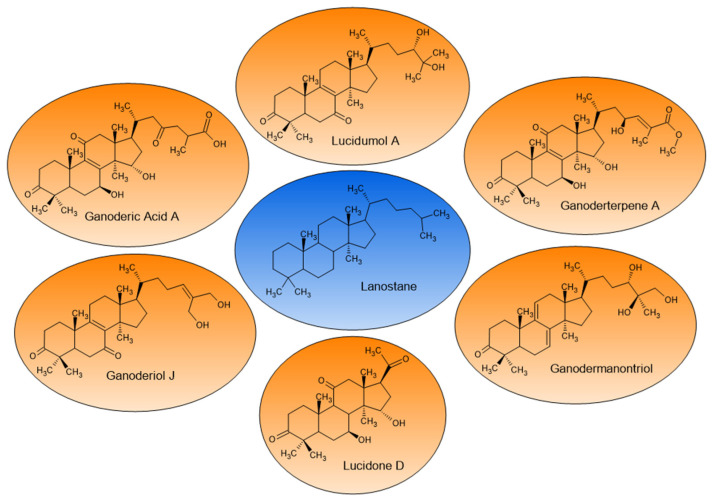
Chemical structures of lanostane, the tetracyclic triterpene, and lanostane-type triterpenes isolated from *G. lucidum*: ganoderic acid A, lucidumol A, ganoderterpene A, ganoderiol J, lucidone D, and ganodermanontriol.

Among the 23 studies, only 4 did not identify the composition of the extracts, and the most frequently identified triterpenoids included the lanostane-type ganoderic and lucidenic acids with their subtypes, as well as esters derived from the corresponding carboxylic acids, such as methyl ganoderate, methyl lucidenate, and ganoderterpene A (Table 1). Furthermore, some *Ganoderma* alcohols, such as ganoderiol, lucidumol, and ganodermanontriol, were commonly identified. The ganoderic acids encountered are named as A, B, C, C1, C2, C5, C6, D, E, F, G, H, K, J, Mh, V, U, α, among others. The chemical structures of some triterpenes extracted from *G. lucidum* are shown in Figure 3.

The most different compounds were characterized by one study [32], that identified sesquiterpenes in the *G. lucidum* triterpenes extract.

### 3.4. Inflammatory Models Investigated In Vitro

Fifteen of the 23 selected articles explored the anti-inflammatory effects of triterpenes using cell culture models (Table 2). The use of cellular lineage models is crucial in the preclinical evaluation of novel molecules and is particularly valuable for understanding the modulation of key inflammatory mediators, including cytokines and chemokines, thus facilitating the identification of promising therapeutic agents that can later progress to in vivo studies [40]. Of the 15 articles that used a cellular model to investigate anti-inflammatory effects, 9 utilized RAW 264.7 cell line. Among these studies, all employed lipopolysaccharide (LPS) to induce inflammation, with the concentration of 1 µg/mL and a stimulation duration of 24 h being the most used parameters.

The RAW264.7 cell line is a macrophage-like cell line derived from BALB/c mouse ascites tumor induced by the Abelson leukemia virus. It has been widely used to evaluate immunomodulatory agents due to its robust inflammatory response to LPS, a Toll-Like Receptor (TLR) 4 agonist, as well as its commercial availability and ease of culturing. Compounds that effectively influence the levels of mediators like IL-6, TNF-α, and NO in RAW264.7 cells are expected to exhibit similar effects in vivo, aiding in the modulation of inflammatory responses. However, the extent to which results obtained from RAW264.7 cells translate to human immune cells remains unclear [41].

Among the six other studies that opted not to use RAW264.7 cells for investigating inflammatory processes, various other cell types were employed, each with distinct methods for inducing inflammation (Table 2). These different models included human nucleus pulposus cells isolated from patients with intervertebral disc degeneration and stimulated with IL-1β; human peripheral blood mononuclear cells (PBMCs) stimulated with LPS or TNF-α; BV-2 murine microglial cell line stimulated with LPS; Human Umbilical Vein Endothelial Cells (HUVECs) stimulated with hydrogen peroxide; NRK-52E cells, derived from rat renal proximal tubule epithelial cells (specific inflammation induction method not reported); Ana-1 macrophage cells stimulated with LPS and D-galactosamine; and finally, the human immortalized keratinocyte line (HaCaT) stimulated with recombinant human IL-17a (rhIL-17a) and recombinant human TNF-α (rhTNF-α).

### 3.5. Anti-Inflammatory Effects of Triterpenes from Ganoderma lucidum in Cells

The treatment concentrations of triterpenes or extracts varied among the studies, but the most used concentrations consisted in a range of 6.25 to 50 µM or 2.5 to 50 µg/mL, being the maximum values tested at 80 µM and 500 µg/mL. The majority of studies observed a dose-dependent anti-inflammatory effect.

Of the 15 studies that employed cellular models to investigate the impact of triterpenes on inflammation, 10 studies assessed nitric oxide (NO) produced, while 7 studies evaluated the expression of inducible nitric oxide synthase (iNOS). iNOS is an enzyme induced by various inflammatory stimuli and catalyzes the production of NO. The expression of iNOS serves as a critical indicator of the cell’s capacity to produce NO. The release of NO can either promote or inhibit inflammation, depending on the context and intensity of the immune response. An imbalance in NO production is associated with various inflammatory diseases.

The other inflammatory markers verified in the studies were the cytokines TNF-α, IL-6 and IL-1β, and the COX-2 enzyme as well as prostaglandin E2 (PGE2) (Table 2). It was observed that in all studies, the treatment with *G. lucidum* triterpenes (isolated or as a mixture) promoted a reduction in the levels or mRNA expression of such pro-inflammatory markers, with no exception. However, the difference among the studies was the composition of the treatment: some studies treated the cells with the crude triterpene extract, while others tested isolated compounds.

Butyl lucidenate D2 was tested in two studies and demonstrated a strong inhibition in NO production reaching 70% compared to the vehicle control [21,22]. Methyl lucidenate L inhibited NO production with an IC_50_ value of 38.6 ± 1.0 μM [1]. A prominent effect was observed by Kou et al. [28], that isolated and tested ganoderterpene A showing an IC_50_ equivalent to 7.15 μM, surpassing the inhibitory activity of the positive control, quercetin (IC_50_ = 9.05 μM). Other compounds such as lucidumol A and ganodermanontriol inhibited NO production by 86.5% and 88.2%, at a concentration of 50 µM, respectively, surpassing L-NMMA’s inhibition (73.6% at 50 μM) [27].

Crude extracts containing triterpenes of *G. lucidum* also presented significant inhibition of TNF-α and IL-6 in a dose-dependent manner, with IC_50_ values of 15.1 and 14.4 μg/mL, respectively [15]. Another study significantly reduced TNF-α levels at concentrations of 50 and 100 μg/mL, with an IC_50_ value of 33.8 μg/mL [30].

### 3.6. Meta-Analysis of Anti-Inflammatory Effects of In Vitro Studies

Nine in vitro studies met the inclusion criteria for the meta-analysis. These studies evaluated the anti-inflammatory effects of triterpenes from *G. lucidum* in LPS- or IL-1β-stimulated macrophages by measuring NO, IL-6, and TNF-α levels. Although outcome data were reported in different units among the studies (e.g., μM or μg/mL), they were consistently expressed as mean ± SD.

#### 3.6.1. Meta-Analysis of NO Measurements

**Figure 4:**
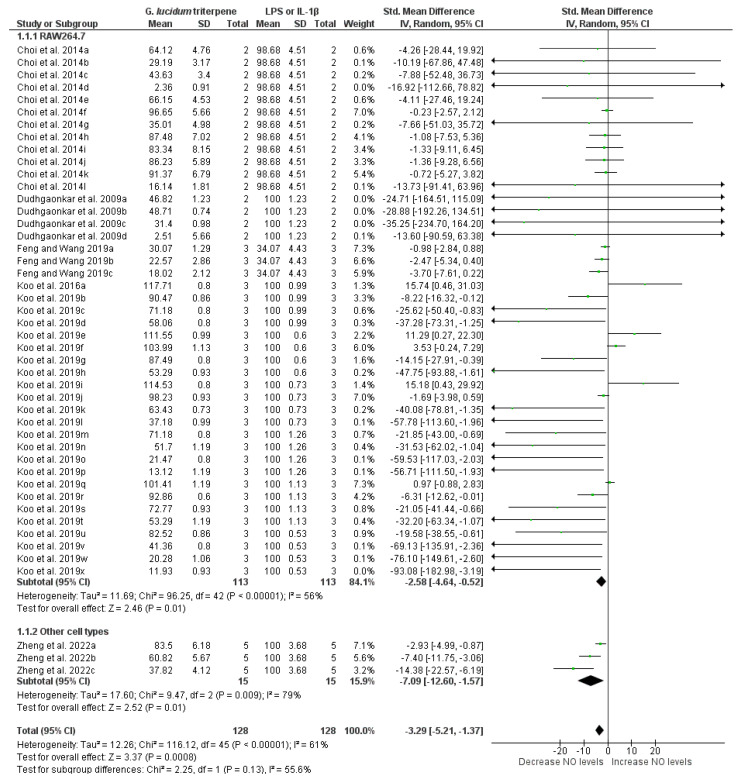
Forest plot of the standardized mean difference in NO levels measured in cells stimulated with LPS or IL-1β followed by treatment with *G. lucidum* triterpenes, at the following concentrations: Choi et al. [22]: 20 µM GT1 (a), 20 µM GT2 (b), 20 µM GT3 (c), 20 µM GT4 (d), 20 µM GT5 (e), 20 µM GT6 (f), 20 µM GT7 (g), 20 µM GT8 (h), 20 µM GT9 (i), 20 µM GT10 (j), 20 µM GT11 (k), and 20 µM GT12 (l); Dudhgaonkar et al. [15]: 3 µg/mL GLT (a), 10 µg/mL GLT (b), 30 µg/mL GLT (c), and 50 µg/mL GLT (d); Feng and Wang [24]: 10 µM LUC (a), 20 µM LUC (b), and 40 µM LUC (c); Koo et al. [27]: 6.25 µM Ganosidone A (a), 12.5 µM Ganosidone A (b), 25 µM Ganosidone A (c), 50 µM Ganosidone A (d), 6.25 µM Methyl ganoderate A (e), 12.5 µM Methyl ganoderate A (f), 25 µM Methyl ganoderate A (g), 50 µM Methyl ganoderate A (h), 6.25 µM Methyl ganoderate H (i), 12.5 µM Methyl ganoderate H (j), 25 µM Methyl ganoderate H (k), 50 µM Methyl ganoderate H (l), 6.25 µM Lucidumol A (m), 12.5 µM Lucidumol A (n), 25 µM Lucidumol A (o), 50 µM Lucidumol A (p), 6.25 µM Ganoderic acid A (q), 12.5 µM Ganoderic acid A (r), 25 µM Ganoderic acid A (s), 50 µM Ganoderic acid A (t), 6.25 µM Ganodermanontriol (u), 12.5 µM Ganodermanontriol (v), 25 µM Ganodermanontriol (w), and 50 µM Ganodermanontriol (x); Zheng et al. [38]: 6.5 µM Ganoderic acid A (a), 12.5 µM Ganoderic acid A (b), and 25 µM Ganoderic acid A (c). Funnel plot is in Supplementary Materials (Figure S1).

Five studies (forty-six groups; two subgroups) reporting NO levels were included in the quantitative synthesis. Overall, our analysis demonstrated that triterpenes from *G. lucidum* significantly reduced NO levels (−3.29 [−5.21, −1.37]; *p* = 0.0008) in cells previously challenged with LPS or IL-1β (Figure 4), despite moderate heterogeneity (I^2^ = 61%). Subgroup analysis revealed no significant difference between groups (Chi^2^ = 2.25; df = 1; *p* = 0.13). Specifically, in RAW264.7 cells, NO levels were significantly decreased compared to untreated cells (−2.58 [−4.64, −0.52]; *p* = 0.01), with moderate heterogeneity (I^2^ = 56%). A significant reduction in NO levels was also observed in other cell types (−7.09 [−12.60, −1.57]; *p* = 0.01) by *G. lucidum* triterpenes, although heterogeneity was high (I^2^ = 79%).

#### 3.6.2. Meta-Analysis of IL-6 Measurements

**Figure 5:**
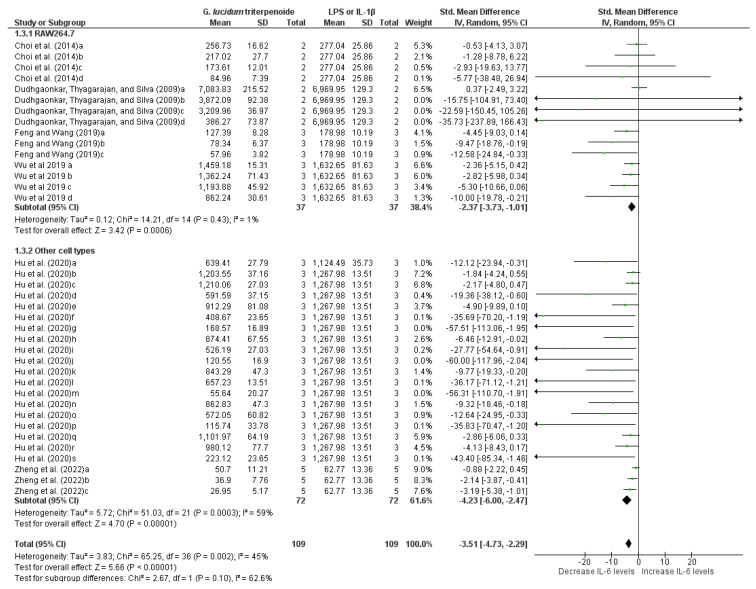
Forest plot of the standardized mean difference in IL-6 levels measured in cells stimulated with LPS or IL-1β followed by treatment with *G. lucidum* triterpenes, at the following concentrations: Choi et al. [22]: 5 µM GT-2 (a), 10 µM GT-2 (b), 25 µM GT-2 (c), and 50 µM GT-2 (d); Dudhgaonkar et al. [15]: 3 µg/mL GLT (a), 10 µg/mL GLT (b), 30 µg/mL GLT (c), and 50 µg/mL GLT (d); Feng and Wang [24]: 10 µM LUC (a), 20 µM LUC (b), and 40 µM LUC (c); Wu et al. [1]: 10 µM Ganoderterpene A (a), 20 µM Ganoderterpene A (b), 40 µM Ganoderterpene A (c), and 80 µM Ganoderterpene A (d); Hu et al. [26]: 25 µg/mL *G. lucidum* ethanol extract (a), 5 µg/mL 20-OH Lucideric A (b), 10 µg/mL 20-OH Lucideric A (c), 25 µg/mL 20-OH Lucideric A (d), 5 µg/mL 20-OH Lucideric N (e), 10 µg/mL 20-OH Lucideric N (f), 25 µg/mL 20-OH Lucideric N (g), 5 µg/mL Ganodermanontriol (h), 10 µg/mL Ganodermanontriol (i), 25 µg/mL Ganodermanontriol (j), 5 µg/mL Ganoderiol A (k), 10 µg/mL Ganoderiol A (l), 25 µg/mL Ganoderiol A (m), 5 µg/mL Ganoderiol F (n), 10 µg/mL Ganoderiol F (o), 25 µg/mL Ganoderiol F (p), 5 µg/mL Ganoderiol D (q), 10 µg/mL Ganoderiol D (r), and 25 µg/mL Ganoderiol D (s); Zheng et al. [38]: 6.5 µM Ganoderic acid A (a), 12.5 µM Ganoderic acid A (b), and 25 µM Ganoderic acid A (c). Funnel plot is in Supplementary Materials (Figure S2).

Six studies (thirty-seven groups; two subgroups) evaluating IL-6 levels were included in the meta-analysis. Overall, triterpenes from *G. lucidum* significantly reduced IL-6 levels (−3.51 [−4.73, −2.29]; *p* < 0.00001) in cells challenged with LPS or IL-1β (Figure 5), with mild heterogeneity (I^2^ = 45%). Subgroup analysis revealed no significant difference between groups (Chi^2^ = 2.67; df = 1; *p* = 0.10). In RAW264.7 cells, a significant reduction in IL-6 levels was observed compared to untreated cells (−2.37 [−3.73, −1.01]; *p* = 0.0006), with low heterogeneity (I^2^ = 1%). Similarly, triterpenes from *G. lucidum* also significantly reduced IL-6 levels in other cell types (−4.23 [−6.00, −2.47]; *p* < 0.00001), despite moderate heterogeneity (I^2^ = 59%).

#### 3.6.3. Meta-Analysis of TNF-α Measurements

**Figure 6:**
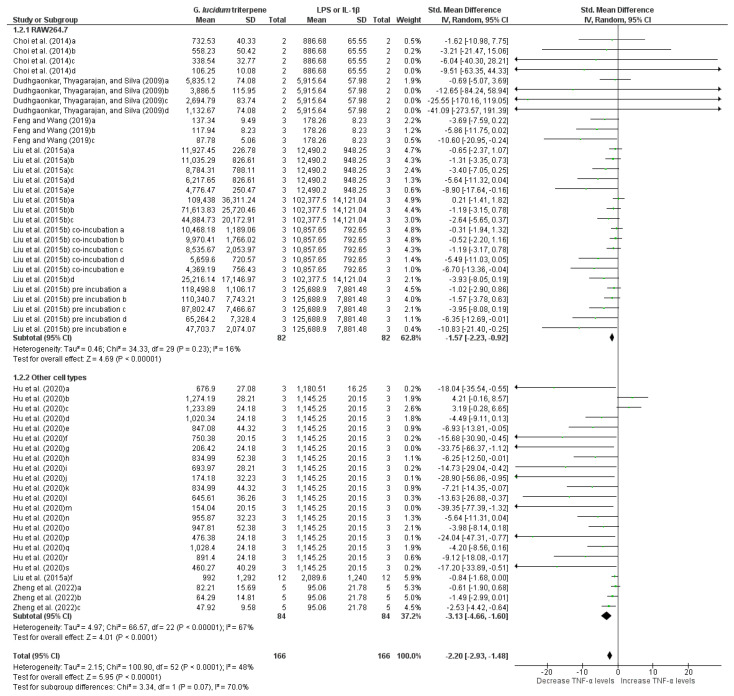
Forest plot of the standardized mean difference in TNF-α levels measured in cells stimulated with LPS or IL-1β followed by treatment with *G. lucidum* triterpenes, at the following concentrations: Choi et al. [22]: 5 µM GT-2 (a), 10 µM GT-2 (b), 25 µM GT-2 (c), and 50 µM GT-2 (d); Dudhgaonkar et al. [15]: 3 µg/mL GLT (a), 10 µg/mL GLT (b), 30 µg/mL GLT (c), and 50 µg/mL GLT (d); Feng and Wang [24]: 10 µM LUC (a), 20 µM LUC (b), and 40 µM LUC (c); Liu et al. (2015a) [16]: 2.5 µg/mL Ganoderic acid C1 (a), 5 µg/mL Ganoderic acid C1 (b), 10 µg/mL Ganoderic acid C1 (c), 20 µg/mL Ganoderic acid C1 (d), 40 µg/mL Ganoderic acid C1 (e), and 20 µg/mL Ganoderic acid C1 (f); Liu et al. (2015b) [30]: 12.5 µg/mL *G. lucidum* (a), 25 µg/mL *G. lucidum* (b), 50 µg/mL *G. lucidum* (c), 40 µg/mL Ganoderic acid C1 (d); co-incubation (e–i): 100 µg/mL *G. lucidum* (e), 2.5 µg/mL Ganoderic acid C1 (f), 5 µg/mL Ganoderic acid C1 (g), 10 µg/mL Ganoderic acid C1 (h), and 20 µg/mL Ganoderic acid C1 (i), as well as pre-incubation (j–n): 2.5 µg/mL Ganoderic acid C1 (j), 5 µg/mL Ganoderic acid C1 (k), 10 µg/mL Ganoderic acid C1 (l), 20 µg/mL Ganoderic acid C1 (m), and 40 µg/mL Ganoderic acid C1 (n); Hu et al. [26]: 25 µg/mL *G. lucidum* ethanol extract (a), 5 µg/mL 20-OH Lucideric A (b), 10 µg/mL 20-OH Lucideric A (c), 25 µg/mL 20-OH Lucideric A (d), 5 µg/mL 20-OH Lucideric N (e), 10 µg/mL 20-OH Lucideric N (f), 25 µg/mL 20-OH Lucideric N (g), 5 µg/mL Ganodermanontriol (h), 10 µg/mL Ganodermanontriol (i), 25 µg/mL Ganodermanontriol (j), 5 µg/mL Ganoderiol A (k), 10 µg/mL Ganoderiol A (l), 25 µg/mL Ganoderiol A (m), 5 µg/mL Ganoderiol F (n), 10 µg/mL Ganoderiol F (o), 25 µg/mL Ganoderiol F (p), 5 µg/mL Ganoderiol D (q), 10 µg/mL Ganoderiol D (r), and 25 µg/mL Ganoderiol D (s); Zheng et al. [38]: 6.5 µM Ganoderic acid A (a), 12.5 µM Ganoderic acid A (b), and 25 µM Ganoderic acid A (c). Funnel plot is in Supplementary Materials (Figure S3).

Seven studies (fifty-three groups; two subgroups) evaluating TNF-α levels were included in the meta-analysis. The overall analysis revealed that triterpenes from *G. lucidum* significantly reduced TNF-α levels (−2.20 [−2.93, −1.48]; *p* < 0.00001) in cells challenged with LPS or IL-1β (Figure 6), with moderate heterogeneity (I^2^ = 70%). Subgroup analysis indicated no significant difference between groups (Chi^2^ = 3.34; df = 1; *p* = 0.07). In RAW264.7 cells, the meta-analysis showed that triterpenes from *G. lucidum* caused a significant decrease in TNF-α levels (−1.57 [−2.23, −0.92]; *p* < 0.00001), with low heterogeneity (I^2^ = 16%). In other cell types, a significant decrease in TNF-α levels compared to controls was also observed (−3.13 [−4.66, −1.60]; *p* < 0.00001), despite moderate heterogeneity (I^2^ = 67%).

### 3.7. Animal Models in Studying Inflammation

Nine of the 23 selected articles explored the anti-inflammatory effects of triterpenes using animal models (Table 3). Regarding these experiments, the majority of studies used mice as animal models (6 studies) due to their ease of handling, low cost, availability of well-characterized strains, and genetic similarities to humans in preclinical research [42]. In addition, 2 studies evaluated the inflammatory effects in chickens, that have a well-characterized immune system, which makes them valuable for researching immune responses, vaccine development, and infectious diseases [43]. And only 2 studies used rats (together with mice models).

Among the 6 articles analyzed, the different strains of mice were: BALB/c, specific pathogen-free ICR and SENCAR, C57BL/6J, Swiss, and Swiss albino mice; while Hyline laying chickens, Hailan white chickens, Wistar and Sprague-Dawley rats were also tested. The latter experimental model was used to evaluate anti-arthritic activity.

In both studies using chickens’ models, the inflammation inducer was cadmium chloride (CdCl_2_). This compound is a toxic heavy metal that induces cellular stress and inflammation by activating the NF-κB and MAPK signaling pathways, leading to an increase in pro-inflammatory cytokines [44]. In the mice models, different inflammation inducers were used, such as 12-*O*-tetradecanoylphorbol-13-acetate (TPA), 5-fluorouracil (5-FU), and a suspension of carrageenan. These compounds induce inflammation through the activation of the immune system and the release of pro-inflammatory mediators [45,46,47]. The treatment concentrations with triterpenes varied among the studies, with doses ranging from 10 mg/kg to 300 mg/kg for mice and rats, while chickens received ~10 mg.

### 3.8. Anti-Inflammatory Effects of Triterpenes from G. lucidum in Animal Models

Although the inflammation was induced by different methods, the anti-inflammatory effect evaluated in the animal models was similar in 6 studies, which measured the production of proteins and/or expression of mRNA related to pro-inflammatory cytokines (TNF-α, IL-6, IL-1β) or the anti-inflammatory cytokine IL-10. It was observed that there was a reduction in TNF-α, IL-6 and IL-1β expression in liver [29] and the levels in spleen [37] of chickens submitted to intoxication by cadmium. Both studies tested the effect of the supplementation with a *G. lucidum* triterpenoids extract, and both found that the treated groups exhibited a significant reduction in the pro-inflammatory cytokines.

An anti-inflammatory effect observed in mice and rats treated with *G. lucidum* triterpene extract was observed when the authors tested three models: inflammation induced by carrageenan, by formalin and by Freund’s complete adjuvant (FCA). The treatment significantly reduced the paw edema caused in all models and this effect was observed in a dose-dependent level. The first model showed a reduction of 76.09% at the dose of 100 mg/kg [35].

Another study investigated the athero-protective effect of a triterpene extract from *G. lucidum,* and the authors observed that orally administered extract protected the carotid artery from disturbed flow-induced atherogenesis. Furthermore, when the extract was administered subcutaneously, it prevented carotid artery ligation-induced neointima formation and also ameliorated the oxidative stress promoted by the atherogenesis induction.

The only study that tested isolated triterpenes from *G. lucidum* in animal models, evaluated ear edema and observed inhibitory doses (ID_50_) ranging from 0.07 to 0.39 mg/ear depending on the compound [20]. Notably, lucidenic acid N demonstrated one of the most potent effects, with an ID_50_ of 0.07 mg/ear. These results were comparable to or even exceeded the anti-inflammatory activity of the reference drug indomethacin (ID_50_ = 0.30 mg/ear). These findings suggest that lucidenic and ganoderic acids from *G. lucidum* possess significant anti-inflammatory potential, offering promising leads for the development of therapeutic agents against inflammation.

Mi et al. and Oluba et al. [31,32] observed significant reduction in the levels of TNF-α and increase in IL-10 in the serum of different strains of mice treated with *G. lucidum* triterpenoids extract. The former study evaluated the inflammation induced by maternal separation stress and tested small doses of treatment (10–40 mg/kg); while the last one evaluated the capacity of the triterpenoids extract to mitigate the inflammatory effects caused by *Plasmodium berghei* infection, using higher doses (100–250 mg/kg). In both studies, the inflammatory balance was restored with triterpenoids treatment (Table 3).

Due to great variability of the experimental models, the in vivo studies were not evaluated by meta-analysis.

Among the 23 selected articles with in vitro and in vivo studies, approximately half of them reported triterpenes showed a relation with the NF-κB pathway by downregulating or inhibiting this gene expression; and five articles observed a relation with both NF-κB and MAPK (Table 4). A study observed that *G. lucidum* triterpenes inhibited the transcription of NF-κB by suppression of p65 phosphorylation and suppressed MAP kinases by down-regulating the phosphorylation of ERK1/2 and JNK, but not p38 [15]. In contrast, another study observed that sterols isolated from the same mushroom restrained the phosphorylation of p38, but not ERK and JNK [48].

Abulizi et al. [19] investigated the effect of Ganoderic Acids (GA) extract on inflammation induced by chemotherapy with 5-fluorouracil (5-FU) in tumor-bearing mice. Co-treatment with GA, administered at a concentration of 50 mg/kg/day, effectively reduced the overexpression of TNF-α, IL-6, IL-1β, iNOS, and COX2. The authors suggested that GA extract exhibited its effects by downregulating the TLR4/Myd88/NF-κB signaling pathway (Table 4).

## 4. Discussion

The objective of this systematic review and meta-analysis was to consolidate the evidence regarding the anti-inflammatory potential of *G. lucidum* triterpenes. The results consistently demonstrated the capacity of these compounds to significantly reduce inflammatory markers in both in vitro and in vivo experimental models.

The mushroom *G. lucidum* has been traditionally used as a co-adjuvant in cancer treatment, most commonly as tincture preparations. Triterpenes, a large and diverse group of natural organic compounds derived from mushrooms, have emerged as promising candidates for the modulation of inflammatory responses. The remarkable structural diversity of triterpenes in *G. lucidum* has led to extensive studies of their pharmacological activities, including their anti-cancer, antioxidant, immunomodulatory and anti-inflammatory properties [7,49,50].

This knowledge has been further strengthened by evidence showing that many *Ganoderma* compounds exhibiting anti-inflammatory effects can be extracted using hydroalcoholic solvents and isolated to be tested in different experimental models. However, the major limitation lies in the extraordinary chemical diversity of triterpenes present in these extracts, with more than 300 compounds identified, which hampers their complete purification and systematic biological evaluation. For instance, Ganoderic Acid A (GA-A) often demonstrates stronger inhibitory activity in models of renal fibrosis and cysts compared to other monomers [34]. While GA-A and GA-B exhibit anti-inflammatory activity through distinct mechanisms of action, GA-C2 has been shown to inhibit DNA fragmentation, reinforcing the complexity of structure-activity relationships within this chemical class. The overall results and the meta-analyses demonstrated that *G. lucidum* triterpenes, whether evaluated as isolated compounds or as crude extracts, are consistently anti-inflammatory. Nevertheless, the effective concentrations reported across studies varied substantially, with IC_50_ values ranging from 7.15 to 38.6 µM for isolated compounds and from 14 to 33.8 µg/mL for crude extracts. This variability is likely attributable to differences in the intrinsic potency of individual triterpenes, such as methyl lucidenate L and ganoderterpene A [1,28]. In addition, crude extracts probably exhibited divergent IC_50_ values because they were prepared using distinct extraction protocols, including ethanol or aqueous extraction followed by partitioning with methylene chloride, resulting in different chemical compositions [15,30]. Consequently, the anti-inflammatory efficacy of these extracts depends directly on both the qualitative and quantitative triterpene profile, making standardization a major obstacle for their translation into evidence-based medicine.

The influence of processing conditions represents an additional and often overlooked gap in the literature. Some authors have shown that mushroom drying methods, such as freeze-drying versus heat-drying, markedly alter the biological profile of *G. lucidum* preparations [33]. While freeze-drying better preserves antioxidant capacity and total ganoderic acid content, thermal drying at 60 °C may enhance specific anti-inflammatory properties [33]. Furthermore, variations in extraction parameters—including methods, solvents, temperature, and extraction time—produce extracts with distinct compound profiles and relative abundances [28]. These findings emphasize the need for standardized processing and extraction protocols, as the “quality” of *G. lucidum* products is not linear and must be aligned with specific therapeutic objectives.

Mechanistically, nuclear factor-κB (NF-κB) and mitogen-activated protein kinases (MAPKs) belong to the core inflammatory signaling pathways that are essential for initiating, amplifying, and modulating inflammatory responses within the immune system [51]. The transcription factor NF-κB plays a fundamental role in regulating genes associated with inflammatory and immune responses, as well as cell survival and death. Its activation can be induced by a wide range of stimuli, including cytokines, pathogen-associated molecular patterns (PAMPs), damage-associated molecular patterns (DAMPs), and reactive oxygen species (ROS) [52]. In parallel, similar stimuli promote the activation of mitogen-activated protein kinase (MAPK) cascades, particularly the JNK and p38 pathways, which often act synergistically to amplify and sustain inflammatory responses. Importantly, most of the studies included in this systematic review reported inhibition, suppression or downregulation of NF-κB and/or MAPK signaling following treatment with *G. lucidum* triterpenes [1,15,16,19,22,23,26,27,28,30,34,37,38]. This modulation was consistently associated with reduced expression and production of pro-inflammatory cytokines, enzymes and nitric oxide (NO) [53]. Another relevant mechanism, independent of NF-κB and MAPK signaling, involves the induction of heme oxygenase-1 (HO-1), an enzyme that plays a key role in maintaining cellular homeostasis and protecting tissues against oxidative and inflammatory damage. The anti-inflammatory effects of HO-1 are largely mediated by its by-products, bilirubin and carbon monoxide, which exert antioxidant and immunomodulatory activities [22]. It has been demonstrated that *Ganoderma* triterpenes, particularly buthyl lucidenate D2, induce HO-1 expression via the PI3K/AKT-Nrf2 pathway. However, the precise molecular determinants underlying this response—such as the most favorable chemical structures or receptor interactions—remain poorly characterized.

In the meta-analysis of in vitro studies, triterpenes from *G. lucidum* consistently attenuated inflammatory responses, regardless of the cell line employed or whether the compounds were tested as isolated molecules or as crude extracts. This finding suggests that anti-inflammatory activity is an intrinsic property of this chemical class, supporting their potential use as alternatives or adjuncts to synthetic non-steroidal anti-inflammatory drugs (NSAIDs). However, despite their promising in vitro potency, *G. lucidum* triterpenes generally exhibit poor oral bioavailability, which represents a major translational limitation. Recent evidence suggests that advanced delivery strategies, such as solid lipid nanoparticles, may be required to enhance their absorption and systemic efficacy [25]. To date, no mushroom-based commercial products have been specifically developed with the aim of improving triterpene bioavailability.

Although the traditional use of *Ganoderma* preparations is supported by experimental studies confirming their anti-inflammatory potential, substantial gaps remain with respect to standardization and pharmacological characterization. In particular, dose–response relationships, long-term toxicity, pharmacokinetics, and bioavailability have not been systematically evaluated, limiting the interpretation of efficacy and safety across studies.

In recent years, a large number of *G. lucidum* dietary supplements have entered the market, driven by growing consumer interest in natural strategies for managing disease and physical or psychological stress. This trend raises critical concerns regarding product quality and regulatory oversight, as such supplements are not consistently evaluated by regulatory agencies and often present labeling inaccuracies. For example, an analysis of 19 *G. lucidum* products commercialized in the United States revealed that only 26.3% were compliant with their label claims [4]. Furthermore, many of these products are introduced into the market without prior safety assessment, thereby increasing potential risks to consumers [4]. Collectively, these findings highlight the urgent need for studies that not only confirm the therapeutic benefits of *Ganoderma* triterpenes, but also establish their effective concentrations, toxicity thresholds, optimal formulations, and appropriate routes of administration to ensure reproducible efficacy and safety.

## 5. Conclusions

This systematic review and meta-analysis synthesized and critically evaluated the current body of evidence regarding the anti-inflammatory properties of triterpenes derived from *Ganoderma lucidum*. Across diverse experimental models, these compounds consistently demonstrated the ability to modulate inflammatory responses, irrespective of whether they were administered as isolated molecules or as components of crude extracts.

Despite this consistent biological signal, significant barriers remain to the translational application of *G. lucidum* triterpenes. The extraordinary structural diversity of these compounds complicates their isolation, identification, and comparative evaluation, often requiring advanced analytical approaches such as high-resolution chromatography coupled to mass spectrometry and nuclear magnetic resonance spectroscopy, which are not universally accessible.

Mechanistic studies largely converge on the inhibition of NF-κB and MAPK signaling pathways; however, additional mechanisms, including HO-1 induction, remain insufficiently explored.

Importantly, the literature still lacks consensus regarding the most potent individual triterpene(s), the relevance of synergistic interactions within complex extracts, and the influence of processing and extraction methods on biological activity. Moreover, fundamental pharmacological aspects—including bioavailability, optimal routes of administration, formulation strategies, and long-term safety—have yet to be adequately addressed. Future research should therefore prioritize standardized processing and extraction protocols, systematic structure–activity relationship studies, and well-designed in vivo investigations integrating pharmacokinetics, toxicity, and efficacy endpoints. Addressing these gaps will be essential for translating the anti-inflammatory potential of *G. lucidum* triterpenes into safe, effective, and evidence-based pharmaceutical or therapeutic applications.

## Figures and Tables

**Table 1:** Extraction and isolation procedures to obtain *G. lucidum* triterpenes.

Bioactive Compound(s)	Extraction Solvent	Extraction Conditions	Isolation Technique	Characterization Technique	Reference
Ganoderic acid extract GA composed of: GA-A (16.1%), GA-B (10.6%), and GA-C2 (5.4%)	Reported in study not available	Reported in study not available	Reported in study not available	HPLC ^1^	[19]
Lucidenic acid B, Methyl lucidenate C, Lucidenic acid F, Lucidenic acid N, Ganoderic acid A, Ganoderic acid C1, Ganoderic acid C2, Ganoderic acid DM, Ganodermanondiol, Ganolactone, Fungisterol, 5,6-Dihydroergosterol, Ergosterol, Ergosterol peroxide, 9(11)-Dehydroergosterol peroxide, Demethylincisterol A3	MeOH	3×, 3 L, 14 days (each), at RT ^2^	Silica gel open-column chromatography followed by preparative reversed-phase HPLC ^1^ with a series of silica C18 columns (25 cm × 10 cm i.d.)	The compounds were identified by comparison of their spectroscopic data with those reported in the literature.	[20]
GT1: Butyl lucidenate E2, GT2: Butyl lucidenate D2, GT3: Butyl lucidenate P, GT4: Butyl lucidenate Q, GT5: ganoderiol F, GT6: Methyl ganodenate H, GT7: Methyl ganodenate J, GT8: Ludidumol B, GT9: Ganodermanondiol, GT10: Methy lucidenate N, GT11: Methy lucidenate A, GT12: Butyl lucidenate N	CHCl_3_-soluble fraction	Reported in previous study [21]	Reported in previous study [21]	Reported in previous study [21]	[22]
Ganodermanontriol, Ganoderiol J, Ganoderiol D, Ganoderiol A, Lucidadiol, Ganoderiol F, Ganoderiol B, Ganoderic acid DM	95% EtOH	Dried powder was soaked in the solvent (1:15, *w*/*v*) for 24 h. Then, it underwent 3 cycles of reflux extraction at 100 °C. The obtained liquid was subsequently filtered, concentrated under reduced pressure and then lyophilized.	HPLC ^1^ was carried out with a diode-array detector (DAD) and C18 spherical columns (5 µm, 4.6 × 250 mm)	LC–MS/MS ^5^	[23]
Triterpene extract (GLT) composed of: Ganoderic acid A, Ganoderic acid F, Ganoderic acid H, Ganoderic acid Mh, Ganoderic acid S1, Ganosporeric acid, Lucidenic acid B, Lucidenic acid D, Lucidenic acid D1, Lucidenic acid E1, Lucidenic acid L, Methyl lucidenate G	95% EtOH	Not described	HPLC ^1^	NMR ^3^, MS ^4^ and LC/MS ^5^	[15]
Lucidone D (LUC)	EtOH	Purchased from Shanghai Yihe Biotechnology Co., Ltd., Shanghai, China	Not described	Not described	[24]
*Ganoderma* triterpenoids (GT) mainly composed of: Ganoderic acid A (21%), Ganoderic acid B (8%), Ganoderic acid C (4%), Ganoderic acid C5 (3%), Ganoderic acid C6 (1%), Ganoderic acid D (10%), Ganoderic acid E (2%), Ganoderic acid G (5.5%), Ganoderenic acid D (7.5%)	Acidic EtOAc solution	Donated from the Biotechnology Research and Development Institute of Double Crane Group, Taiwan	Not described	Reversed-phase HPLC ^1^	[25]
*G. lucidum* ethanol extract and 20-OH Lucideric A or 20-OH Lucideric N or Ganodermanontriol or Ganoderiol A or Ganoderiol F, or Ganoderiol D	CHCl_3_	3×, 3 h	silica gel column and silica gel chromatography	UHPLC-MS ^6^	[26]
Ganosidone A, Methyl ganoderate A, Methyl ganoderate H, Lucidumol A, Lucidumol C, Ganoderic acid A, Ganodermanontriol Ganolucidic acid A, Ganolucidic acid E	MeOH	3×, 2 L at RT ^2^	Partition with *n*-hexane, EtOAc and *n*-butanol. EtOAc extracts were subjected to silica gel chromatography, Sephadex LH-20 gel column and semi-preparative HPLC ^1^	IR ^7^, UV ^8^, NMR ^3^, HRESIMS ^9^	[27]
Ganoderterpene A, Methyl ganoderate A, Methyl ganoderate C, Lucidumol A, Ganodertriol M, Ganoderiol J, Ganodermanondiol, Lucidumol B, Ganodermanontriol, (24R,25S)-24,25,26-trihydroxy-lanosta-7,9(11)-dien-3-one, 6-deshydrocerevisterol, 6-O-methyl-cerevisterol, 5α-ergosta-7,22-dien-3β-ol	Pretreated with MeOH, and the residue was extracted with EtOH, giving the extract EA	Pretreatment: 6×, 12 h Extraction: 10×	With CHCl_3_:CH_3_OH (*v*/*v*, 100:1 to 1:1) the extract EA provided six fractions, that were purified by different methods (silica gel column, HPLC ^2^, Sephadex LH-20 chromatography)	NMR ^3^, UV ^8^, HRESIMS ^9^	[28]
*G. lucidum* triterpenoids	Not described	The dried *G. lucidum* was pulverized by a Chinese medicine grinder into a powder.	TLC ^10^ and preparative HPLC ^1^	Oleanolic acid was used as a standard to quantify the total triterpenoids in the extract.	[29]
Ganoderic acid C1	Aqueous extract was partitioned with methylene chloride	Not described	Fractionated and purified using repeated silica gel, preparative HPLC ^1^, and Sephadex LH-20 column chromatography	NMR ^3^ and LC-MS ^5^	[16]
Triterpenoid-enriched MC fraction composed of: Ganoderiol F, Ganoderic acid α, Ganoderic acid V, Ganoderic acid H, Ganoderic acid C2, Ganolucidic acid A, Ganolucidic acid E, Ganoderic acid C1, Ganoderenic acid A, Ganolucidic acid D, Ganoderic acid U, Ganoderic acid J, Ganoderic acid A, Ganoderic acid K Ganoderenic acid D	Aqueous extract was partitioned with methylene chloride	Not described	Fractionated and purified using repeated silica gel, preparative HPLC ^1^, and Sephadex LH-20 column chromatography	NMR ^3^ and LC-MS ^5^	[30]
*G. lucidum* triterpenoids extract (GLTs)	EtOH 95%	50 L, at RT ^2^	The extract was partitioned with EtOAc and the soluble fraction was separated by D101 macroporous resin and eluted with MeOH/H_2_O, producing four fractions. F1 extract was purified by silica gel column chromatography	TLC ^10^ and LC-MS ^5^	[31]
Ganoderma terpenoid extract (GTE) composed of: Component 1: [(2Z,4E)-6-[(3S,4R,5S,6R,7Z,10S)-4,10-dihydroxy-6-(3-hydroxypropyl)-10-methyl-7-(1-oxopropan-2-ylidene)spiro[4.5]decan-3-yl]-2-(4-methylpent-3-enyl)hepta-2,4,6-tri-enyl] acetate, Component 2: 3-[18-(2-carboxyethyl)-8,13-bis(1-hydroxyethyl)-3,7,12,17-tetramethyl-22,23-dihydroporphyrin-2-yl]propanoic acid, Component 3: (2R)-2-[(3S,5R,10S,13R,14R,16R,17R)-3-acetyloxy-16-hydroxy-4,4,10,13,14-pentamethyl-2,3,5,6,7,11,12,15,16,17-decahydro-1H-cyclopenta[a]phenanthren-17-yl]-6-methyl-5-methylideneheptanoic acid, Component 4: (6E,8E,10E,12E,14E,16E,18E,20E,22E,24E,26E)-2,6,10,14,19,23,27,31-octamethyldotriaconta-6,8,10,12,14,16,18,20,22,24, 26,30-dodecaen-2-ol, Component 5: 1-[2,6-dinitro-4-(trifluoromethyl)phenyl]-2-[6-methyl-4-(trifluoromethyl)pyridin-2-yl]hydrazine, Component 6: 2-(hydrox-ymethyl)-6-[[19-methoxy-8-[(E)-6-methoxy-6-methylhept-4-en-2-yl]-5,9,17,17-tetramethyl-18-oxapentacyclo [10.5.2.01,13.04,12.05,9]nonadec-2-en-16-yl]oxy]oxane-3,4,5-triol, Component 7: tungsten, dicarbonyl-(g-4–2-methylenecycloheptanone)[1,2-bis(dimethylphosphino)ethane, Component 8: (7S)-2-(cycloheptylamino)-11-(4-methyl-1,4-diazepane-1-carbonyl)-7-propan-2-yl-5,7-dihydrobenzimidazolo[1,2-d][1,4]benzodiazepin-6-one, Component 9: (2E,6E,8E,10E,12E,14E,16E,18E,20E,22E,24E,26E)-2,6,10,14,19,23,27,31-octamethyldotriaconta-2,6,8,10,12,14,16,18,20,22,24,26,30-tridecaen-1-ol, Component 10: 5-[(1R,2S,4R,6R,7R,10S,11R,14S,16S)-14,16-dihydroxy-7,11-dimethyl-3-oxapentacyclo [8.8.0.02,4.02,7.011,16]octadecan-6-yl]pyran-2-one, Component 11: (1S,2R,3R,4S,5R,6S,8R,9R,13S,16S,17R,18S)-11-ethyl-4,6,18-trimethoxy-13-(methoxymethyl)-11-azahexacyclo [7.7.2.12,5.01,10.03,8.013,17]nonadecane-8,9,16-triol	50% EtOH	Extraction: 1 h	Partition: CHCl_3_/H_2_O (1:1, *v*/*v*, 3×). Further extraction with saturated NaHCO_3_ (3×), followed by acidification with 6 N HCl to a pH of 3–4. The solution was then extracted with EtOAc (3×)	GC-MS ^11^	[32]
Ganoderic acids extracts composed of: Ganoderic acid eta, Butylganoderate A, Ganoderic acid C2, Ganoderic acid A, Lucidenic acid N, Ganoderic acid G, Ganoderenic acid B, Ganoderic acid B, Ganoderenic acid K, Ganoderic acid K, Lucidenic acid A, Ganoderenic acid D, Ganoderic acid D, Ganoderic acid H, 20-hydroxynganoderic acid AM1, Ganoderic acid C6, Lucidenic acid D, Ganoderic acid F, 12-acetyl ganoderic acid F	EtOH	Extraction: 0.1 g/mL at 50 °C, 60 °C and 70 °C, for 1, 2 or 3 h	The ganoderic acid extract was tested as crude extract.	UHPLC/Q-TOF-MS ^12^ and comparison of their MS ^4^ spectra with those reported in the literature	[33]
Ganoderic acids extract composed of three main monomers: GA-A (16.1%), GA-B (10.6%), and GA-C2 (5.4%)	H_2_O	Not described	Alcohol precipitation method from the extract-like product precipitated at the bottom of concentration tank during water extraction of *G. lucidum*	HPLC ^1^	[34]
Total triterpene extract	EtOH	Not described	Solubilization with chloroform followed by elution through silica gel column and elution with petroleum ether, chloroform, methanol and combinations of these solvents.	Not described	[35]
Ganoluciduone A, Ganoluciduone B, Ganolucidoid A, Ganolucidoid B, (24S, 25R)-25-Methoxylanosta-7,9(11)-dien-3β,24,26-triol, 26,27-Dihydroxy-24,25-epoxylanosta-7,9(11)-dien-3-one, 3β-hydroxy-12β-acetoxy-7,11,15,23-tetraoxolanosta-8,20E(22)-dien-26-oic acid methyl ester, 15α-hydroxy-3,11,23-trioxolanosta-8,20E(22)-dien-26-oic acid methyl ester	MeOH	3×, under reflux	Partition with EtOAc, and the EtOAc extract was purified by silica gel and Sephadex LH-20 column chromatography; preparative TLC ^10^; RP-HPLC ^13^	IR ^7^, UV ^8^, NMR ^3^	[36]
*G. lucidum* triterpenoids	EtOH	Extraction proportion (1:20), 80 °C for 3 h	Partition with CHCl_3_, 3×, followed by extraction with saturated sodium hydrogen carbonate, adjustment of pH with HCl and extraction with CHCl_3_.	Colorimetric measurement compared with oleanolic acid, as standard.	[37]
Butyl lucidenate P, Butyl lucidenate E2, Butyl lucidenate D2, Butyl lucidenate Q, 5,6-dihydro-ergosterol, Ergosterol, Ganoderiol F, Methyl ganoderate H, Ergosterol peroxide, Methyl ganoderate J, Lucidumol B, Ganodermanondiol, Methyl lucidenate N, Methyl lucidenate A, Butyl lucidenate N	CHCl_3_-soluble fraction	Not described	Repeated column chromatography	The compounds were identified by comparing the physicochemical and spectroscopic data (IR ^7^, UV ^8^, MS ^4^, NMR ^3^) with those reported in the literature.	[21]
12β-Acetoxy-3β,28-dihydroxy-7,11,15,23-tetraoxo-5α-lanosta-8-en-26-oic acid, Lucidenic acid R Methyl lucidenate K Methyl lucidenate L 7β,15α,20-Trihydroxy-3,11,23-trioxo-5α-lanosta-8-en-26-oic acid, 12β-Acetoxyganoderic acid θ, Methyl ganoderate C1, 12-acetoxyganoderic acid D, Methyl ganoderate F, Ganoderic acid E, Methyl ganoderate E, Ganoderic acid F, Ganoderic acid C, Methyl ganoderate C, Ganoderic acid J, Methyl lucidenate D2, Methyl lucidenate A, Ganoderenic acid C, Ganoderenic acid A, Methyl lucidenate H, Ganoderenic acid K, 12β-acetoxy-7β-hydroxy-3,11,15,23-tetraoxo-5α-lano-sta-8,20-dien-26-oic acid, 12β-acetoxy-3,7,11,15,23-pentaoxo-lanosta-8,20-dien-26-oic acid, Ganoderenic acid B, Ganoderenic acid G, Methyl ganoderenate D, Methyl ganoderate P, Ganoderenic acid F, Ganoderenic acid D, Ganoderic acid η, Ganoderic acid ζ, Lucidone F, Ganoderic acid I	80% EtOH	9 kg of sliced fruiting bodies; 90 L; 2 h; 2×	Partition with H_2_O, cyclohexane, EtOAc, *n*-butanol. Silica gel column chromatography and preparative HPLC ^1^	IR ^7^, UV ^8^, TLC ^10^, NMR ^3^, HRESIMS ^9^	[1]
Ganoderic acid A (GAA)	Not described	Not described	Not described	Not described	[38]

^1^ HPLC: High-performance liquid chromatography; ^2^ RT: Room temperature; ^3^ NMR: Nuclear Magnetic Resonance; ^4^ MS: Mass spectrometry; ^5^ LC-MS: Liquid chromatography coupled to mass spectrometry; ^6^ UHPLC-MS: Ultra-high performance liquid chromatography coupled to mass spectrometry; ^7^ IR: Infrared spectroscopy; ^8^ UV: Ultraviolet spectroscopy; ^9^ HRESIMS: High-resolution electrospray ionization mass spectrometry; ^10^ TLC: Thin-layer chromatography; ^11^ GC-MS: Gas-chromatography coupled to mass spectrometry; ^12^ UHPLC/Q-TOF-MS: Ultra-high performance liquid chromatography-quadrupole time-of-flight mass spectrometry; ^13^ RP-HPLC: Reversed-phase high-performance liquid chromatography. Solvents abbreviations: methanol (MeOH), ethanol (EtOH), ethyl acetate (EtOAc), chloroform (CHCl_3_), hydrogen chloride (HCl).

**Table 2:** Anti-inflammatory activity of *G. lucidum* triterpenes in vitro.

Bioactive Compound(s)	Cell Line Model	Inflammation Induction	Treatment Concentration	Effect	Reference
GT1: Butyl lucidenate E2, GT2: Butyl lucidenate D2, GT3: Butyl lucidenate P, GT4: Butyl lucidenate Q, GT5: ganoderiol F, GT6: Methyl ganodenate H, GT7: Methyl ganodenate J, GT8: Ludidumol B, GT9: Ganodermanondiol, GT10: Methy lucidenate N, GT11: Methy lucidenate A, GT12: Butyl lucidenate N	RAW264.7 cells	LPS (1 µg/mL)	20 µM	↓ NO levels	[22]
GT-2: Butyl lucidenate D2			0–50 µM	↓ NO, TNF-α and IL-6 levels ↓ iNOS and COX-2 expression	
Triterpene extract (GLT)	RAW264.7 cells	LPS (1 μg/mL)	10–50 μg/mL	↓ NO, TNF-α, IL-6 and PGE2 levels ↓ COX-2 and iNOS expression	[15]
Lucidone D (LUC)	RAW264.7 cells	LPS (1 μg/mL)	10, 20, and 40 μM	↓ NO, TNF-α and IL-6 levels ↓ COX-2 and iNOS expression	[24]
Ganoderma triterpenoids (GT)	Human umbilical vein endothelial cells (HUVECs)	Laminar shear stress (LSS, 12 dyn/cm^2^), or oscillatory shear stress (OSS, ±5 dyn/cm^2^), or static control for 24 h using the ibidi pump system	500 μg/mL	↓ induction of V-CAM-1, TNF-α, IL-6 expression	[25]
*G. lucidum* ethanol extract	Ana-1 cells	LPS (1 μg/mL)	25 μg/mL	↓ NO, TNF-α, IL-6, IL1β and PGE2 levels	[26]
20-OH Lucideric A or 20-OH Lucideric N or Ganodermanontriol or Ganoderiol A or Ganoderiol F, or Ganoderiol D			5, 10 and 25 μg/mL	↓ NO, TNF-α, IL-6, IL1β and PGE2 levels	
Ganosidone A, Methyl ganoderate A, Methyl ganoderate H, Lucidumol A, Lucidumol C, Ganoderic acid A, Ganodermanontriol, Ganolucidic acid A, Ganolucidic acid E	RAW264.7 cells	LPS (0.5 ng/mL)	6.25; 12.5; 25 e 50 μM	↓ NO levels	[27]
Ganoderterpene A, Methyl ganoderate A, Methyl ganoderate C, Lucidumol A, Ganodertriol M, Ganoderiol J, Ganodermanondiol, Lucidumol B, Ganodermanontriol, (24R,25S)-24,25,26-trihydroxy-lanosta-7,9(11)-dien-3-one, 6-deshydrocerevisterol, 6-O-methyl-cerevisterol, and 5α-ergosta-7,22-dien-3β-ol	BV-2 microglial cells	LPS (1 μg/mL)	20 μM	↓ NO levels	[28]
Ganoderterpene A			25 μM	↓ iNOS, TNF-α, COX-2, IL-1β and IL-6 expression	
Ganoderic acid C1	RAW 264.7 cells	LPS (1 μg/mL)	2.5, 5, 10, 20, 40 μg/mL	↓ TNF-α levels	[16]
	PBMCs	LPS (2 mg/mL)	20 μg/mL		
*G. lucidum* extract	RAW 264.7 cells LPS (1 μg/mL)		12.5, 25, 50, and 100 μg/mL	↓ TNF-α levels	[30]
Triterpenoid-enriched MC fraction			25 μg/mL	↓ TNF-α levels	
Ganoderiol F, Ganoderic acid α, Ganoderic acid V, Ganoderic acid H, Ganoderic acid C2, Ganolucidic acid A, Ganolucidic acid E, Ganoderic acid C1, Ganoderenic acid A, Ganolucidic acid D, Ganoderic acid U, Ganoderic acid J, Ganoderic acid A, Ganoderic acid K, Ganoderenic acid D			10 and 20 μg/mL	↓ TNF-α levels	
Ganoderic acid C1			2.5, 5, 10, 20 and 40 μg/mL	↓ TNF-α levels	
Ganoderic acid C1	PBMCs	LPS (2 μg/mL)	20 μg/mL	↓ TNF-α levels	
Ganoderic acids extracts	HaCaT cells	rhIL-17A (50 ng/mL) and rhTNF-α (10 ng/mL)	10 μg/mL	↓ IL-6 expression	[33]
Ganoderic acids extract composed of three main monomers: GA-A, GA-B and GA-C2	NRK-52E cells	hypoxia/reoxygenation (H/R) model	3.125, 12.5, and 50 µg/mL	↓ COX-2, iNOS and IL-6 levels	[34]
Ganoluciduone A, Ganoluciduone B, Ganolucidoid A, Ganolucidoid B, (24S, 25R)-25-Methoxylanosta-7,9(11)-dien-3β,24,26-triol, 26,27-Dihydroxy-24,25-epoxylanosta-7,9(11)-dien-3-one, 3β-hydroxy-12β-acetoxy-7,11,15,23-tetraoxolanosta-8,20E(22)-dien-26-oic acid methyl ester, 15α-hydroxy-3,11,23-trioxolanosta-8,20E(22)-dien-26-oic acid methyl ester	RAW264.7 cells	LPS (1 μg/mL)	50 μM	↓ NO levels	[36]
Ganoluciduone B			12.5 μM	↓ NO levels	
Butyl lucidenate P, Butyl lucidenate D2, Butyl lucidenate Q, Ergosterol peroxide, Methyl ganoderate J, Butyl lucidenate N	RAW264.7 cells	LPS (1 μg/mL)	50 μM	↓ NO levels	[21]
Butyl lucidenate P or Butyl lucidenate D2 or Butyl lucidenate N			1, 3, 10, and 30 μg/mL	↓ NO levels; ↓ COX-2 and iNOS expression	
33 *G. lucidum* isolated triterpenoids	RAW264.7 cells	LPS (1 μg/mL)	50 μM	↓ NO levels	[1]
Methyl Lucidenate L			10, 20, 40 and 80 μM	↓ IL-1β, IL-6 and NO levels ↓ iNOS and COX-2 expression	
Ganoderic acid A (GAA)	human nucleus pulposus (NP) cells	IL-1β (10 ng/mL)	6.25, 12.5, and 25 μM	↓ NO, PGE2, TNF-α and IL-6 levels ↓ TNF-α, IL-6, COX-2 and iNOS expression	[38]

↓ Reduction.

**Table 3:** Anti-inflammatory activity of *G. lucidum* triterpenes in vivo.

Bioactive Compound(s)	Animal Model	Inflammation Induction	Treatment Concentration	Effect	Reference
Ganoderic acid extract GA composed of: GA-A (16.1%), GA-B (10.6%), and GA-C2 (5.4%)	Female BALB/c mice (6–7 weeks)	5-FU (30 mg/kg)	GA (50 mg/kg)	↓ TNF-α, IL-6, IL-1β, iNOS and COX-2 expression	[19]
Lucidenic acid A *, Methyl lucidenate A *, Lucidenic acid D2 *, Methyl lucidenate D2 *, Lucidenic acid E2 *, Methyl lucidenate E2 *, Lucidenic acid P *, Methyl lucidenate Q *, 20-Hydroxylucidenic acid N *, Ganoderic acid A, Ganoderic acid F *, Ganoderic acid DM, Ganoderic acid T-Q *	Specific pathogen-free female ICR and SENCAR mice	A solution of 1.7 nmol 12-*O*-tetradecanoylphorbol-13-acetate (TPA) (1 µg in 20 mL acetone)	Each compound was tested in two concentrations: 0.5 mg/ear and 1.0 mg/ear	Edema reduction comparable or better than indomethacin	[20]
Ganodermanontriol	Sprague–Dawley rats	LPS (5 mg/kg)	25, 50 or 100 mg/kg	effectively reversed LPS-induced pulmonary edema in a dose-dependent manner substantial alleviation of interstitial thickening and lung edema ↓ TNF-α, IL-6 and IL-1β expression and levels	[23]
Ganoderma triterpenoids (GT) mainly composed of: Ganoderic acid A (21%), Ganoderic acid B (8%), Ganoderic acid C (4%), Ganoderic acid C5 (3%), Ganoderic acid C6 (1%), Ganoderic acid D (10%), Ganoderic acid E (2%), Ganoderic acid G (5.5%), Ganoderenic acid D (7.5%)	Male BALB/c mice (2–5 months)	Neointima formation induction through a ligature at the end of the left common carotid artery (LCA) near the carotid bifurcation	300 mg/kg/day (orally and subcutaneous injection)	resistance against ligation-induced intimal thickening, ↓ endothelial apoptosis, ↓ oxidative stress ↓ recruitment of monocytes/macrophages to the neointima region	[25]
*G. lucidum* triterpenoids	Hyline male laying chickens (7 day-old)	A diet of 140 mg/kg of CdCl_2_	0.5 mL (20 mg/mL)	↓ TNF-α, IL-1β and IL-6 expression	[29]
*G. lucidum* triterpenoids extract (GLTs)	Adult C57BL/6J mice	Maternal separation for 4 h/day; and sub-threshold variable stress (STVS) by forced swimming in icy water (three times with a one-hour interval in between), tail suspension (for 1 h), and restraint (in a 50 mL conical tube for 1 h)	10, 20 or 40 mg/kg (i.p. injection for 3 weeks)	↓ IL-1β, IL-6, TNFα and ↑ IL-10 mRNA expression and protein levels	[31]
Ganoderma terpenoid extract (GTE)	Male Swiss mice (7 ± 1.2 weeks)	Mice were inoculated with 0.2 × 10^5^/mL *Plasmodium berghei*-infected erythrocytes suspended in phosphate-buffered saline	100 mg/kg GTE and 250 mg/kg GTE	↓ TNF-α and ↑ IL-10 levels	[32]
Total triterpene extract	Male Swiss albino mice (6 weeks) used for anti-inflammatory studies and female Wistar rats (15 weeks) used for anti-arthritic studies	-Acute inflammation was produced in Male Swiss albino mice by the sub-plantar injection of 20 µL of freshly prepared 1% suspension of carrageenan in saline on the right hind paw. -Chronic Paw edema was produced in Male Swiss albino mice by 20 µL of freshly prepared 2% formalin. -Arthritis was induced by the intradermal injection of 0.1 mL of Freund’s Complete Adjuvant (FCA) into the sub-planar region of the right hind paw.	10, 50, and 100 mg/kg body weight total triterpene extract	↓ paw edema in models induced by carrageenan, formalin, and FCA.	[35]
*G. lucidum* triterpenoids	Hailan white chickens (7-day-old)	A diet of 140 mg/kg of CdCl_2_	0.5 mL of Ganoderma lucidum triterpenoid solution (20 mg/mL) per day	↓ TNF-α, IL-1β and IL-6 levels	[37]

* Compounds isolated in previous study of the group. ↓ Reduction. ↑ Increase.

**Table 4:** Mechanisms of action of anti-inflammatory activity proposed for *G. lucidum* triterpenes.

Bioactive Compound(s)	Mechanisms of Action	Reference
In vitro experiments		
GT1: Butyl lucidenate E2, GT2: Butyl lucidenate D2, GT3: Butyl lucidenate P, GT4: Butyl lucidenate Q, GT5: ganoderiol F, GT6: Methyl ganodenate H, GT7: Methyl ganodenate J, GT8: Ludidumol B, GT9: Ganodermanondiol, GT10: Methy lucidenate N, GT11: Methy lucidenate A, GT12: Butyl lucidenate N	Induction HO-1 expression via the PI3K/AKT-Nrf2 pathway, independent of MAPK or antioxidant defenses signaling	[22]
GT-2: Butyl lucidenate D2		
Triterpene extract (GLT)	Suppression of NF-κB and AP-1 signaling pathways, along with inhibition of MAP kinases (ERK1/2 and JNK)	[15]
Lucidone D (LUC)	-	[24]
Ganoderma triterpenoids (GT)	-	[25]
*G. lucidum* ethanol extract	Inhibition of TLR4-MyD88-mediated NF-κB and MAPK signaling pathways	[26]
20-OH Lucideric A or 20-OH Lucideric N or Ganodermanontriol or Ganoderiol A or Ganoderiol F, or Ganoderiol D		
Ganosidone A, Methyl ganoderate A, Methyl ganoderate H, Lucidumol A, Lucidumol C, Ganoderic acid A, Ganodermanontriol, Ganolucidic acid A, Ganolucidic acid E	Inhibition NF-κB signaling pathway	[27]
Ganoderterpene A, Methyl ganoderate A, Methyl ganoderate C, Lucidumol A, Ganodertriol M, Ganoderiol J, Ganodermanondiol, Lucidumol B, Ganodermanontriol, (24R,25S)-24,25,26-trihydroxy-lanosta-7,9(11)-dien-3-one, 6-deshydrocerevisterol, 6-O-methyl-cerevisterol, and 5α-ergosta-7,22-dien-3β-ol	Suppressed the activation of MAPK and TLR-4/NF-κB signaling pathways	[28]
Ganoderterpene A		
Ganoderic acid C1	Downregulation of the NF-κB signaling pathway	[16]
*G. lucidum* extract	Suppression of the NF-κB, AP-1 and MAPK pathways	[30]
Triterpenoid-enriched MC fraction		
Ganoderiol F, Ganoderic acid α, Ganoderic acid V, Ganoderic acid H, Ganoderic acid C2, Ganolucidic acid A, Ganolucidic acid E, Ganoderic acid C1, Ganoderenic acid A, Ganolucidic acid D, Ganoderic acid U, Ganoderic acid J, Ganoderic acid A, Ganoderic acid K, Ganoderenic acid D		
Ganoderic acid C1		
Ganoderic acids extracts	-	[33]
Ganoderic acids extract composed of three main monomers: GA-A, GA-B and GA-C2	Downregulating TLR4/MyD88/NF-κB signaling pathway	[34]
Ganoluciduone A, Ganoluciduone B, Ganolucidoid A, Ganolucidoid B, (24S, 25R)-25-Methoxylanosta-7,9(11)-dien-3β,24,26-triol, 26,27-Dihydroxy-24,25-epoxylanosta-7,9(11)-dien-3-one, 3β-hydroxy-12β-acetoxy-7,11,15,23-tetraoxolanosta-8,20E(22)-dien-26-oic acid methyl ester, 15α-hydroxy-3,11,23-trioxolanosta-8,20E(22)-dien-26-oic acid methyl ester	-	[36]
Ganoluciduone B		
Butyl lucidenate P, Butyl lucidenate D2, Butyl lucidenate Q, Ergosterol peroxide, Methyl ganoderate J, Butyl lucidenate N	-	[21]
Butyl lucidenate P or Butyl lucidenate D2 or Butyl lucidenate N		
33 *G. lucidum* isolated triterpenoids	Reduction in NF-κB expression levels	[1]
Methyl Lucidenate L		
Ganoderic acid A (GAA)	Suppression of NF-κB pathway	[38]
In vivo experiments		
Ganoderic acid extract GA composed of: GA-A (16.1%), GA-B (10.6%), and GA-C2 (5.4%)	Inhibition of TLR4/Myd88/NF-κB pathway Downregulated the expression of p-AMPK/AMPK	[19]
Lucidenic acid A Methyl lucidenate A Lucidenic acid D2 Methyl lucidenate D2 Lucidenic acid E2 Methyl lucidenate E2 Lucidenic acid P Methyl lucidenate Q 20-Hydroxylucidenic acid N Ganoderic acid A Ganoderic acid F Ganoderic acid DM Ganoderic acid T-Q	-	[20]
Ganodermanontriol	Inhibition of the activation of the NF-κB and MAPK signaling pathways	[23]
Ganoderma triterpenoids (GT) mainly composed of: Ganoderic acid A (21%), Ganoderic acid B (8%), Ganoderic acid C (4%), Ganoderic acid C5 (3%), Ganoderic acid C6 (1%), Ganoderic acid D (10%), Ganoderic acid E (2%), Ganoderic acid G (5.5%), Ganoderenic acid D (7.5%)	-	[25]
*G. lucidum* triterpenoids	-	[29]
*G. lucidum* triterpenoids extract (GLTs)	-	[31]
*Ganoderma* terpenoid extract (GTE)	-	[32]
Total triterpene extract	-	[35]
*G. lucidum* triterpenoids	Inhibition of NF-κB signaling pathway	[37]

## Data Availability

Data are contained within the article and Supplementary Materials.

## Supplementary Materials

The following supporting information can be downloaded at: https://www.mdpi.com/article/10.3390/ph19010188/s1, Table S1: Studies excluded from meta-analysis evaluation and their reasons.; Figure S1: Funnel plot of the levels of NO observed in the studies included in the meta-analysis; Figure S2: Funnel plot of the cytokine IL-6 activity of the studies included in the meta-analysis; Figure S3: Funnel plot of the cytokine TNF-α activity of the studies included in the meta-analysis. Reference [54] is cited in the Supplementary Materials.
