# Supplementary material for: Anti-Inflammatory Potential of Ganoderma lucidum Triterpenes: A Systematic Review and Meta-Analysis of Preclinical Evidence
Source: Pharmaceuticals (Basel). 2026 Jan 21;19(1):188. doi: 10.3390/ph19010188 (PMC12845357; doi:10.3390/ph19010188)
Supplement: Supplementary file 1 [file pharmaceuticals-19-00188-s001.zip › pharmaceuticals-4046317-supplementary.pdf]

Table S1 - Studies excluded from meta-analysis evaluation and their reasons.

Study	Meta-analysis exclusion reason
**NO evaluation**	
Hu et al., 2020	NO (nitric oxide) production was assessed by measuring nitrite, which was different from the measurements used in the other studies
Kou et al., 2021	NO (nitric oxide) production was assessed by measuring nitrite, which was different from the measurements used in the other studies
Su et al., 2020	Absence of control (vehicle) group
Tung et al., 2013	Measurement based only on the IC50 value, different from the other studies
Wu et al., 2019	Absence of control (vehicle) group and/or indirect evaluation of NO by the expression of iNOS
**IL-6 evaluation**	
Hsu et al., 2018	It was determined only the gene expression
Kou et al., 2021	It was determined only the gene expression
Ryu et al., 2021	It was determined only the gene expression
Shao et al., 2021	IL-6 was measured by Western blot and the graph was converted to "relative level", different from the other measurement that were demonstrated in "pg/mL"
**TNF-α evaluation**	
Hsu et al., 2018	It was determined only the gene expression
Kou et al., 2021	It was determined only the gene expression

Figure S1 – Funnel plot of the levels of NO observed in the studies included in the meta-analysis.

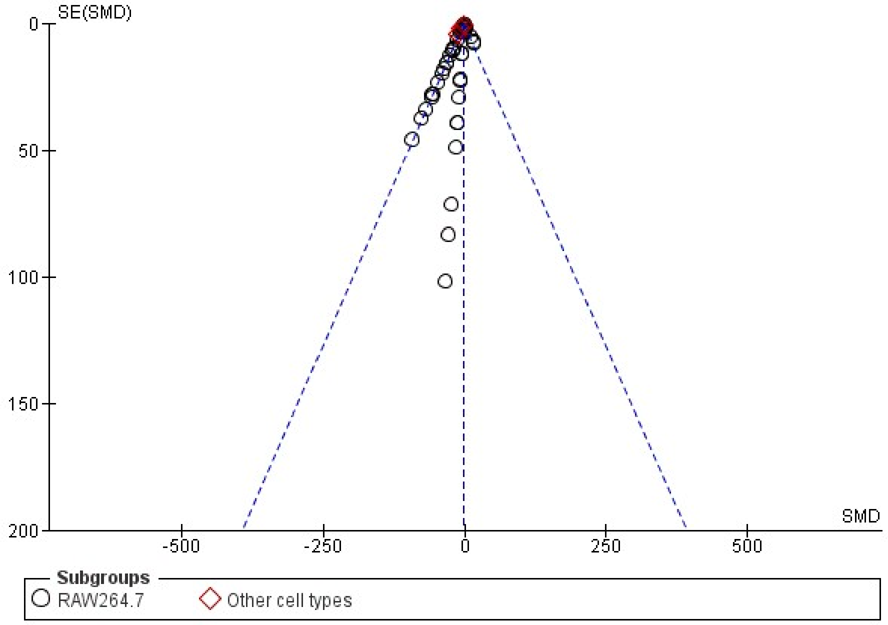

Figure S2 – Funnel plot of the cytokine IL-6 activity of the studies included in the meta-analysis.

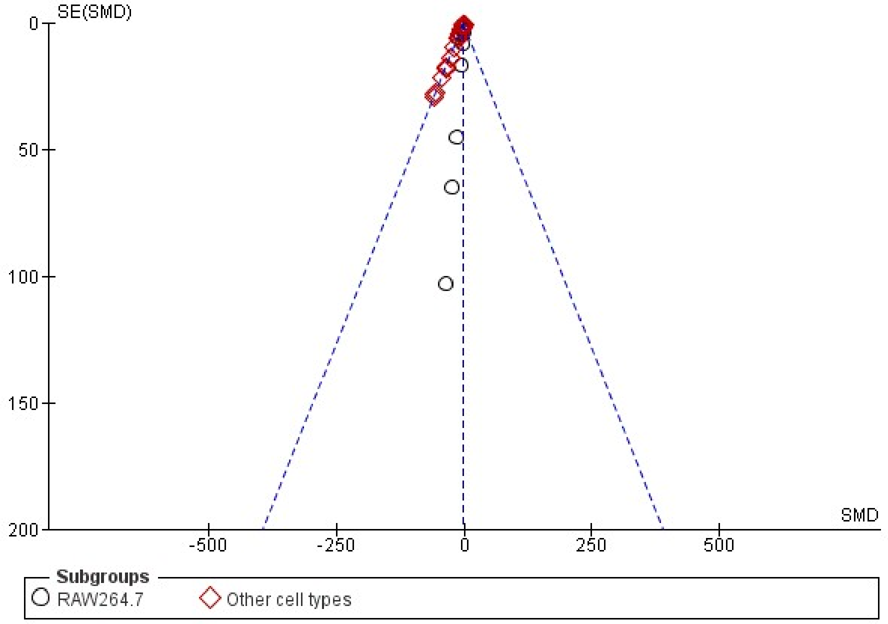

Figure S3 – Funnel plot of the cytokine TNF-α activity of the studies included in the meta-analysis.

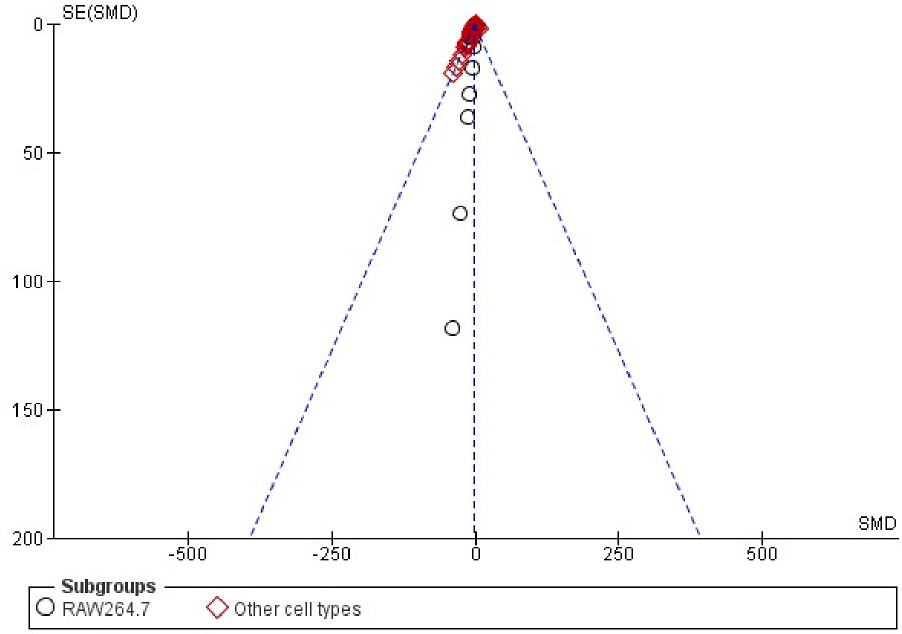

PRISMA 2020 for Abstracts Checklist

Section and Topic	Item #	Checklist item	Reported (Yes/No)
**TITLE**			
Title	1	Identify the report as a systematic review.	p. 2, line 21
**BACKGROUND**			
Objectives	2	Provide an explicit statement of the main objective(s) or question(s) the review addresses.	p. 2 lines 21-23
**METHODS**			
Eligibility criteria	3	Specify the inclusion and exclusion criteria for the review.	p. 2 lines 26-30
Information sources	4	Specify the information sources (e.g. databases, registers) used to identify studies and the date when each was last searched.	p. 2 line 24 and 29
Risk of bias	5	Specify the methods used to assess risk of bias in the included studies.	p. 2 lines 31-32
Synthesis of results	6	Specify the methods used to present and synthesise results.	p. 2 lines 26-32
**RESULTS**			
Included studies	7	Give the total number of included studies and participants and summarise relevant characteristics of studies.	p. 2 lines 33-40
Synthesis of results	8	Present results for main outcomes, preferably indicating the number of included studies and participants for each. If meta-analysis was done, report the summary estimate and confidence/credible interval. If comparing groups, indicate the direction of the effect (i.e. which group is favoured).	p. 2 lines 40-45
**DISCUSSION**			
Limitations of evidence	9	Provide a brief summary of the limitations of the evidence included in the review (e.g. study risk of bias, inconsistency and imprecision).	p. 2 lines 46-50
Interpretation	10	Provide a general interpretation of the results and important implications.	p. 2 lines 46-47
**OTHER**			
Funding	11	Specify the primary source of funding for the review.	Not applicable
Registration	12	Provide the register name and registration number.	p. 2 line 31

PRISMA 2020 for Abstracts Checklist

From: Page MJ, McKenzie JE, Bossuyt PM, Boutron I, Hoffmann TC, Mulrow CD, et al. The PRISMA 2020 statement: an updated guideline for reporting systematic reviews. BMJ 2021;372:n71. doi: 10.1136/bmj.n71. This work is licensed under CC BY 4.0. To view a copy of this license, visit https://creativecommons.org/licenses/by/4.0/

PRISMA

PRISMA 2020 Checklist

Section and Topic	Item #	Checklist item	Location where item is reported
**TITLE**			
Title	1	Identify the report as a systematic review.	p. 1, lines 1-2
**ABSTRACT**			
Abstract	2	See the PRISMA 2020 for Abstracts checklist.	p. 2, lines 19-50
**INTRODUCTION**			
Rationale	3	Describe the rationale for the review in the context of existing knowledge.	p. 4, lines 100-109
Objectives	4	Provide an explicit statement of the objective(s) or question(s) the review addresses.	p. 4, lines 100-109
**METHODS**			
Eligibility criteria	5	Specify the inclusion and exclusion criteria for the review and how studies were grouped for the syntheses.	p. 5, lines 136-151
Information sources	6	Specify all databases, registers, websites, organisations, reference lists and other sources searched or consulted to identify studies. Specify the date when each source was last searched or consulted.	p. 5, lines 136-151
Search strategy	7	Present the full search strategies for all databases, registers and websites, including any filters and limits used.	p. 5, lines 124-134
Selection process	8	Specify the methods used to decide whether a study met the inclusion criteria of the review, including how many reviewers screened each record and each report retrieved, whether they worked independently, and if applicable, details of automation tools used in the process.	p. 4-5, lines 117-122 and lines 124-134
Data collection process	9	Specify the methods used to collect data from reports, including how many reviewers collected data from each report, whether they worked independently, any processes for obtaining or confirming data from study investigators, and if applicable, details of automation tools used in the process.	p. 5-6, line 134 and lines 153-162
Data items	10a	List and define all outcomes for which data were sought. Specify whether all results that were compatible with each outcome domain in each study were sought (e.g. for all measures, time points, analyses), and if not, the methods used to decide which results to collect.	p. 6, lines 156-161
	10b	List and define all other variables for which data were sought (e.g. participant and intervention characteristics, funding sources). Describe any assumptions made about any missing or unclear information.	p. 6, lines 156-161
Study risk of bias assessment	11	Specify the methods used to assess risk of bias in the included studies, including details of the tool(s) used, how many reviewers assessed each study and whether they worked independently, and if applicable, details of automation tools used in the process.	p. 6, lines 164-175
Effect measures	12	Specify for each outcome the effect measure(s) (e.g. risk ratio, mean difference) used in the synthesis or presentation of results.	p. 6, lines 171-175
Synthesis methods	13a	Describe the processes used to decide which studies were eligible for each synthesis (e.g. tabulating the study intervention characteristics and comparing against the planned groups for each synthesis (item #5)).	p. 5-6, p. 21, p. 34
	13b	Describe any methods required to prepare the data for presentation or synthesis, such as handling of missing summary statistics, or data conversions.	p. 5-6, p. 21, p. 34
	13c	Describe any methods used to tabulate or visually display results of individual studies and syntheses.	Tables 1, 2,

Section and Topic	Item #	Checklist item	Location where item is reported
			3, 4
	13d	Describe any methods used to synthesize results and provide a rationale for the choice(s). If meta-analysis was performed, describe the model(s), method(s) to identify the presence and extent of statistical heterogeneity, and software package(s) used.	p. 6-7, lines 177-192
	13e	Describe any methods used to explore possible causes of heterogeneity among study results (e.g. subgroup analysis, meta-regression).	Out of scope of this systematic review
	13f	Describe any sensitivity analyses conducted to assess robustness of the synthesized results.	Out of scope of this systematic review
Reporting bias assessment	14	Describe any methods used to assess risk of bias due to missing results in a synthesis (arising from reporting biases).	Out of scope of this systematic review
Certainty assessment	15	Describe any methods used to assess certainty (or confidence) in the body of evidence for an outcome.	Out of scope of this systematic review
**RESULTS**			
Study selection	16a	Describe the results of the search and selection process, from the number of records identified in the search to the number of studies included in the review, ideally using a flow diagram.	p.7-8, lines 196-206 and Fig. 1
	16b	Cite studies that might appear to meet the inclusion criteria, but which were excluded, and explain why they were excluded.	Out of scope of this systematic review
Study characteristics	17	Cite each included study and present its characteristics.	Tables 1, 2, 3
Risk of bias in studies	18	Present assessments of risk of bias for each included study.	p. 8-9, lines 211-222 and Fig. 2
Results of individual studies	19	For all outcomes, present, for each study: (a) summary statistics for each group (where appropriate) and (b) an effect estimate and its precision (e.g. confidence/credible interval), ideally using structured tables or plots.	Out of scope of this systematic review

Section and Topic	Item #	Checklist item	Location where item is reported
Results of syntheses	20a	For each synthesis, briefly summarise the characteristics and risk of bias among contributing studies.	Out of scope of this systematic review
	20b	Present results of all statistical syntheses conducted. If meta-analysis was done, present for each the summary estimate and its precision (e.g. confidence/credible interval) and measures of statistical heterogeneity. If comparing groups, describe the direction of the effect.	p. 28, 29, 30, 31, 32, 33, 34
	20c	Present results of all investigations of possible causes of heterogeneity among study results.	Out of scope of this systematic review
	20d	Present results of all sensitivity analyses conducted to assess the robustness of the synthesized results.	Out of scope of this systematic review
Reporting biases	21	Present assessments of risk of bias due to missing results (arising from reporting biases) for each synthesis assessed.	Out of scope of this systematic review
Certainty of evidence	22	Present assessments of certainty (or confidence) in the body of evidence for each outcome assessed.	Out of scope of this systematic review
**DISCUSSION**			
Discussion	23a	Provide a general interpretation of the results in the context of other evidence.	p. 46-47, line 527-584
	23b	Discuss any limitations of the evidence included in the review.	p. 48, lines 593-599
	23c	Discuss any limitations of the review processes used.	None
	23d	Discuss implications of the results for practice, policy, and future research.	p. 47, 48, lines 587-603
**OTHER INFORMATION**			
Registration and protocol	24a	Provide registration information for the review, including register name and registration number, or state that the review was not registered.	p. 4, line 115

PRISMA 2020 Checklist

Section and Topic	Item #	Checklist item	Location where item is reported
	24b	Indicate where the review protocol can be accessed, or state that a protocol was not prepared.	p. 4, lines 114-115
	24c	Describe and explain any amendments to information provided at registration or in the protocol.	None
Support	25	Describe sources of financial or non-financial support for the review, and the role of the funders or sponsors in the review.	None
Competing interests	26	Declare any competing interests of review authors.	The authors have no competing interests
Availability of data, code and other materials	27	Report which of the following are publicly available and where they can be found: template data collection forms; data extracted from included studies; data used for all analyses; analytic code; any other materials used in the review.	None

From: Page MJ, McKenzie JE, Bossuyt PM, Boutron I, Hoffmann TC, Mulrow CD, et al. The PRISMA 2020 statement: an updated guideline for reporting systematic reviews. BMJ 2021;372:n71. doi: 10.1136/bmj.n71. This work is licensed under CC BY 4.0. To view a copy of this license, visit https://creativecommons.org/licenses/by/4.0/
